# Central Dental Association of Northern New Jersey

**Published:** 1892-04

**Authors:** 


### CENTRAL DENTAL ASSOCIATION OF NORTHERN NEW JERSEY.

*The President.*—Gentlemen, allow me to introduce the speaker of the evening, Dr. Ottolengui, of New York, who will give us a paper entitled "Pulp-Stones."

(For Dr. Ottolengui's paper, see page 248.)

*The President.*—The paper is now open for discussion.

*Dr. Mills.*—I think this society ought to congratulate itself on having such an able paper read before it.

Dr. Ottolengui has given us the results of his own observations. We are beginning to realize that we are specialists. Such a paper shows the value of scientific research.

*Dr. Watkins.*—The matter of "pulp-stones" has been of considerable interest to me of late.

When I was in Boston attending the Union Meeting last October, I heard a very interesting paper read by Dr. Cook, showing the formations which Dr. Ottolengui has so ably described. A great many of them were the regular pulp-stones, and others were produced by granulations forming on the sides of the pulp-canals. . . . The gentleman who read the paper above alluded to, stated that he had examined over five thousand pulps, and more than forty per cent. of them had some form of calcification. I never looked for pulp-stones before, consequently saw but few. Since then I have found many of the formations which Dr. Ottolengui has mentioned.

I want to emphasize the fact that the treatment of pulps with arsenic, wherever there are pulp-stones, has a tendency to create inflammation, and cause almost unbearable pain.

I remember one case where I treated a lady twenty-eight years of age. I cleaned out the cavity and put in arsenic, but the pain was so severe that I was compelled to remove the arsenic and put in an anodyne. I did this several times, and each time that I applied the arsenic there was great suffering.

After several efforts I succeeded in exposing the pulp and destroying it, but not without a great deal of discomfort to the patient. I removed the pulp, and several pulp-stones with it. After the canals were cleansed, and filled with a temporary filling, there was no pain at all. At length this filling was removed, and the canals were filled with gutta-percha, when the pain started again. The patient suffered intensely. I removed the gutta-percha, and the pain disappeared. I again filled with gutta-percha, and the pain returned. Finally, I suggested to the patient that I remove the tooth. I then cleansed the canals and filled them perfectly to the ends, and removed the ends of the roots which were exostosed, then replanted the tooth. There was a certain amount of irritation, and it continued perhaps three months, but since then—it is now about six months, I think—there has been no return of pain, and the tooth appears to be very solid in the jaw.

*Dr. Sanger.*—Some points about this paper are new to me, and, doubtless, to many of us. The subject of pulp-stones and their devitalization is a matter of great interest; the existence of pulp-stones where there is no apparent exposure; where the dentine is in apparently good condition; where no disease is indicated.

Now, in my own practice, when a patient comes to me with a pulp exposed I do not use arsenic. But in order to devitalize when pulp-stones are present, arsenic is not a prompt and effective agent according to this paper; that is, as I understand it. Now, how are we going to determine when we are to use the arsenic?

*Dr. Luckey.*—There is not much that I have to say on this subject, and find only one or two points as to the correctness of his work, and the question of policy as to when and how to use arsenic.

In most of the cases that Dr. Ottolengui has given us, his patient returned after two or three hours of intense suffering, caused by the inflammation arising from the use of arsenic. I think that if he had used the preparation of arsenic at the latter end of the day instead of in the morning, the patient would not have suffered less, but he would have been less annoyed. His mistake was in putting it in in the morning instead of in the afternoon. If, instead of a combination, he had used a chemically pure preparation of arsenic, his patient would probably have had a hard night, but in the morning, pulp-stones or no pulp-stones, he would have found his patient a great deal better.

*Dr. Watkins.*—If Dr. Luckey were to leave the arsenic in his patient's tooth for forty-eight hours, he would find him still suffering at the end of that time.

*Dr. Evans.*—I would ask if it is necessary to destroy the pulp if there is no pulpitis indicated? When we know that pulp-stones are producing pulpitis, then the case would call for its destruction. Why is it that arsenic causes pain when the pulp is not exposed, and it does not cause it when it is exposed? When a tooth is examined and it is determined it has pulp-stones, what then should the dentist do? If he applies arsenic, he increases the irritation and produces a condition of thrombosis. When a pulp is not exposed, it is confined in a cavity where it cannot expand, the blood-vessels become enlarged, and every pulsation of the heart is felt in that cavity. When a pulp is exposed and arsenic is applied, it is instantly absorbed and devitalization ensues. When you aim to destroy a pulp, and there is a condition of pulpitis, the best thing to do with it is to expose and rupture it; the bleeding will afford relief, and your arsenic will be immediately absorbed. But if I found a person using arsenic on an unexposed pulp in a state of inflammation, I should condemn the treatment. Dr. Watkins says if you apply arsenic to a pulp inflammation will follow. I heard the paper of Dr. Cook, of Boston, in relation to this subject of pulp-stones, and I was surprised to find that such a large percentage was found.

It is degeneration of the pulp that causes pulp-stones. It is a condition of calcification that is slowly progressive. No pulp can be capped that is exposed and the lining membrane is ruptured, as the odontoblasts at that point are atrophied and means of calcification lost.

*Dr. Luckey.*—Every gentlemen here knows that it is important to apply arsenic with discretion. If you apply it once, you may take it out as soon as you please, but your result will be the same,—you will have a destroyed pulp.

I have practised dentistry pretty nearly twenty years, and I do not know of any remedy that has given me more satisfaction than arsenic, and I do not know of more than two or three cases that have been in any way unfavorable in their results in which the arsenic has not done its work, pulp-stones or no pulp-stones.

*Dr. Meeker.*—I suppose a good many of my friends here know that I have always been opposed to the use of arsenic. I have not used it, nor have I even had a particle of it in my office for the past five years. There are only two cases of pulps that I can remember of having destroyed by the application of either chloroform or cocaine within a year.

I go to the meetings and I hear what dentists have to say about the use of arsenic. Possibly I do spend a little more time by my methods, but I don't destroy pulps if there be any possibility of saving them. I do not care how long I am doing my work, so long as the patient is able to stand it.

Where there are cases of pulp-stones, I have never saved a pulp.

*Dr. Mills.*—I do not suppose I have applied arsenic five times in a year, yet I want to say that if it is used intelligently and with discrimination, it is of advantage in superficial decay.

I have a tooth of my own that was treated with arsenic by Dr. Wm. H. Allen. When I went to him for advice, he said, "If you want a good, substantial tooth [my teeth are dense and very well organized], there will be no danger in my using arsenic." He made the application, and a few hours afterwards he removed the softened portion and polished. That tooth is here for any man to see, and is alive.

At the Dental Convention in Saratoga, some years since, this subject was discussed, and among other things it was said, that if a man understands the intelligent use of arsenic, there are circumstances in which it can be safely applied. I do not speak of this as an advocate of the common use of this agent, but only of its limited application. I am perfectly willing to accord to every man the right to his own opinion, and I am also willing to stand on my own record. Dr. Meeker made a statement in opposition to the use of arsenic which was just as dogmatic as mine, and both can be true. I claim that the pulp in my mouth that was exposed and was treated with arsenic is alive. My statement has been corroborated by such men as Dr. Atkinson and others who have been in practice fifty years.

*Dr. Ottolengui.*—I think it must be fifty years since Dr. Atkinson used arsenic for that purpose.

*Dr. Mills.*—I do not think it is courtesy to doubt my statement. I am, as I have already said, perfectly willing to stand on my record.

*Dr. Watkins.*—I first want to say that I hope every young man present was asleep when Dr. Mills made his statement, for I am afraid it may have a bad effect, and some one may go on and use arsenic. I hope they will not. I think the time has gone by for its use for the purpose of destroying sensitive dentine.

*Dr. Ottolengui.*—I do not think that the use of arsenic is ever an advantage, and I think the dentist who uses it will live to regret it. How any one can get up and say openly, in the presence of young men, that arsenic is ever excusable in a tooth that is sensitive, I cannot understand. If you would seek authority for such usage, you would have to go back to the practice of fifty years ago.

Now, this is the idea I wish to impress upon you,—persistent pain; pain that you cannot stop means pulp-stones, and it means it always. There is no question about it.

